# Loss of GGN Leads to Pre-Implantation Embryonic Lethality and Compromised Male Meiotic DNA Double Strand Break Repair in the Mouse

**DOI:** 10.1371/journal.pone.0056955

**Published:** 2013-02-22

**Authors:** Duangporn Jamsai, Anne E. O’Connor, Kathleen D. DeBoer, Brett J. Clark, Stephanie J. Smith, Catherine M. Browne, Jonathan G. Bensley, Julie A. Merriman, Wai Shan Yuen, Peter Koopman, Keith T. Jones, Moira K. O’Bryan

**Affiliations:** 1 Department of Anatomy and Developmental Biology, Monash University, Victoria, Australia; 2 Australian Research Council Centre of Excellence in Biotechnology and Development, Monash University, Victoria, Australia; 3 Institute for Molecular Bioscience, The University of Queensland, St Lucia, Queensland, Australia; 4 School of Biomedical Sciences and Pharmacy, University of Newcastle, Callaghan, NSW, Australia; McGill University, Canada

## Abstract

The integrity of male germ cell genome is critical for the correct progression of spermatogenesis, successful fertilization, and proper development of the offspring. Several DNA repair pathways exist in male germ cells. However, unlike somatic cells, key components of such pathways remain largely unidentified. Gametogenetin (GGN) is a testis-enriched protein that has been shown to bind to the DNA repair protein FANCL via yeast-two-hybrid assays. This finding and its testis-enriched expression pattern raise the possibility that GGN plays a role in DNA repair during spermatogenesis. Herein we demonstrated that the largest isoform GGN1 interacted with components of DNA repair machinery in the mouse testis. In addition to FANCL, GGN1 interacted with the critical component of the Fanconi Anemia (FA) pathway FANCD2 and a downstream component of the BRCA pathway, BRCC36. To define the physiological function of GGN, we generated a *Ggn* null mouse line. A complete loss of GGN resulted in embryonic lethality at the very earliest period of pre-implantation development, with no viable blastocysts observed. This finding was consistent with the observation that *Ggn* mRNA was also expressed in lower levels in the oocyte and pre-implantation embryos. Moreover, pachytene spermatocytes of the *Ggn* heterozygous knockout mice showed an increased incidence of unrepaired DNA double strand breaks (DSBs). Together, our results suggest that GGN plays a role in male meiotic DSB repair and is absolutely required for the survival of pre-implantation embryos.

## Introduction

Faithful preservation of genome integrity in response to intrinsic and extrinsic genotoxic insults is of key importance for gametogenesis [1]. In the male, failed or improper repair of DNA damage can lead to spermatogenic failure, apoptosis and male infertility [2], [3]. Moreover, unrepaired DNA can lead to many types of genetic alterations which may be passed onto the offspring [1].

In addition to extrinsic factors, male germ cell genome is constantly being challenged during normal physiological process in the testis including DNA double strand breaks (DSBs) that occur in spermatocytes during meiosis [4]. The formation and repair of meiotic DSBs is a pivotal process that drives genetic diversity. Meiotic DSBs are induced in a controlled manner, by the action of a type II DNA topoisomerase-like enzyme SPO11 [5]. Once synapsis is complete, DSBs must be repaired to allow the progression of meiosis. Meiotic DSB repair requires both meiosis-specific and ubiquitously expressed proteins. Such proteins act to stabilise and/or recruit other proteins to sites of DSB and to facilitate DSB repair via homologous recombination (HR) [4], [6]. Defects in this process can lead to infertility and increased rates of aneuploidy [6]. Furthermore, gamete aneuploidy can result in embryonic death or developmental defects in the offspring [6]. Many of the precise mechanism of meiotic DSB repair are unknown.

*Ggn* is a male germ cell-enriched gene that encodes multiple alternatively spliced transcripts [7]. In the mouse and human, three conserved protein isoforms, GGN1, GGN2 and GGN3, have been predicted [7]–[9]. We have shown that the largest isoform, GGN1, is localised in spermatocytes, spermatids and ultimately became localised to the sperm tails in the mouse and human testes [8], [9]. GGN1 binds to testis-enriched proteins including CRISP2 [8], OAZ3 and GGNBP1 [10], suggesting the role for GGN1 in spermatogenesis and male fertility.

GGN1 and GGN3 have been shown via yeast-two-hybrid assays to interact with FANCL (Fanconi anemia complementation group L) [7]. FANCL (alias POG) is an E3 ligase ubiquitinating enzyme and is a key component of the DNA interstrand crosslink repair complex known as the Fanconi Anemia (FA) pathway [11]. FA is a genetically heterogeneous genome instability disorder characterised by progressive bone marrow failure, cancer predisposition, congenital abnormalities and infertility [12]. The majority of FA cases are caused by mutations in any one of the 14 FA genes (*FANCA*, -*B*, -*C*, -*D1*, -*D2*, -*I*, -*E*, -*F*, -*G*, -*J*, -*L*, -*M*, -*N* and -*P*) [13], [14]. FANC proteins act as either signal transducers or DNA-processing molecules to facilitate DNA repair. The identification of GGN as a FANCL binding partner and its enriched localisation in spermatocytes raises the possibility that GGN has a role in DSB repair during meiosis.

In this study, we used *in vitro* and *in vivo* models to define the function of GGN. We demonstrated that the largest isoform GGN1 interacts with DNA repair proteins FANCL, FANCD2 and BRCC36 (BRCA1/BRCA2-containing complex, subunit 3, alias BRCC3) in the mouse testis. Loss of GGN results in death of embryos prior to blastocyst stage and compromised DSB repair during male meiosis.

## Results and Discussion

### GGN1 Interacts with Components of DNA Repair Machinery in the Mouse Testis

**Figure 1:**
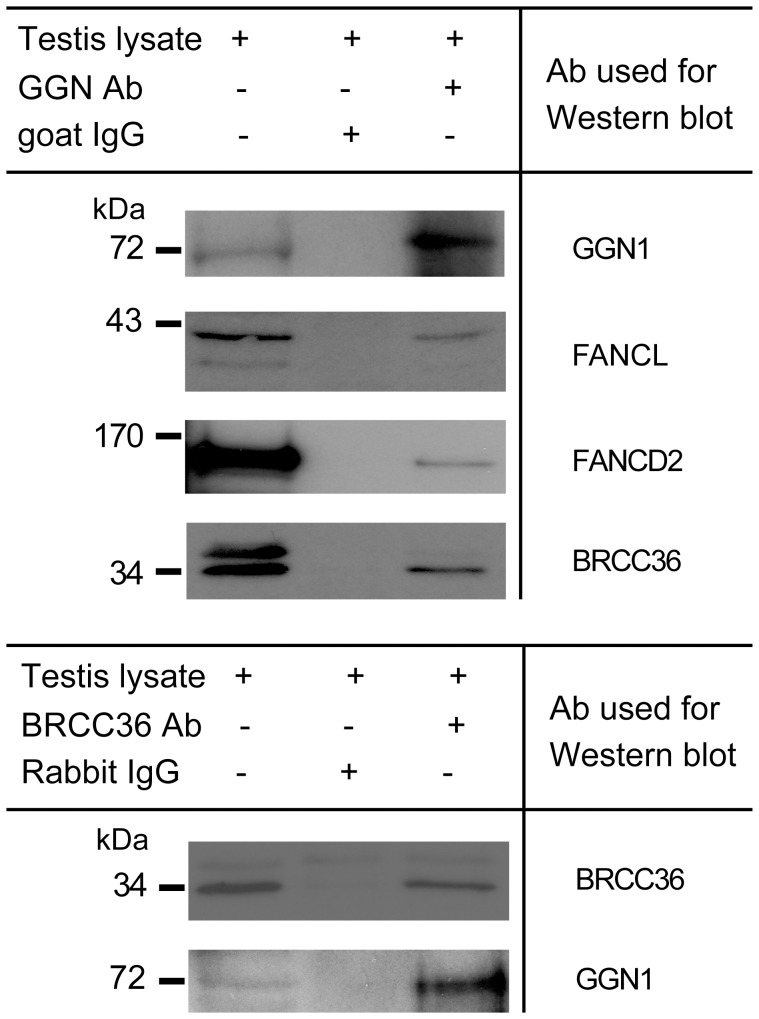
GGN1 is a binding partner of FANCL, FANCD2 and BRCC36 in the mouse testis. (**A**) GGN1 interacted with FANCL, FANCD2 and BRCC36 in the mouse testis as determined by immunoprecipitation using spermatocyte-enriched postnatal day 20 testis lysate. (**B**) GGN1 co-immunoprecipitated with BRCC36 as determined by reciprocal pull down.

GGN1 and GGN3 were previously identified through yeast-two-hybrid assays as a FANCL binding partner [7]. We have previously shown that the largest isoform GGN1 strongly localised in spermatocytes in both mouse and human testes [8], [9] thus we asked if GGN plays a role in DSB repair upon the completion of meiotic recombination. To explore this in more detail, we utilised immunoprecipitation (IP) as a tool to determine endogenous GGN1-interacting partners in the mouse testis. In order to enrich the spermatocyte population and to avoid the presence of post-meiotic germ cells, postnatal day 20 testis was chosen for IPs. Equal amounts of testis extract were loaded onto a GGN1 Ig column and a control goat IgG column. Columns were prepared and used in an identical manner and equal quantities of eluate were loaded onto the SDS-PAGE gels prior to immunoblotting. We began by confirming that endogenous GGN1 bound to FANCL in the testis (Figure 1A), consistent with previous yeast-two-hybrid and over-expression coupled with pull-down studies [7]. This result identifies for the first time that GGN binds to FANCL under normal physiological conditions *in vitro*.

FANCL is a member of the FA core complex that composed of at least 8 proteins including FANCA, -B, -C, -E, -F, -G, -L and -M, with FANCL serving as catalytic subunit [11], [15]. In somatic cells, a primary function of the core complex is to ubiquitinate FANCD2 and FANCI, which in turn leads to a nuclear translocation of the ubiquitinated FANCD2/FANCI complex and the recruitment of several proteins to the sites of DNA damage including proteins within the BRCA pathway. In germ cells, the components of the FA and BRCA pathways and their regulation are largely unidentified. As such, we next investigated if GGN1 also interacted with other components of the FA and BRCA pathways. Elutes from GGN1 testis immunopreciptations were probed with additional antibodies against proteins within the FA and BRCA pathways including FANCA, FANCD2, FANCI, BRCA1 and BRCC36. In addition to FANCL, the major component of the FA pathway, FANCD2, and the deubiquitinating enzyme within the BRCA pathway, BRCC36 [16] (Figure 1A) were co-immunoprecipitated with GGN1 while FANCA, FANCI and BRCA1 were not. The specificity of the interaction was further confirmed by reciprocal IPs. GGN1 was co-immunoprecipitated with BRCC36 (Figure 1B).

In HeLa cells BRCC36 plays a role in DSB repair in response to ionizing radiation and the G2/M cell cycle checkpoint [17], [18]. As a binding partner of FANCL, FANCD2 and BRCC36, we propose that GGN1 plays a role in DNA repair in the testis via its connection with the FA and BRCA pathways.

### Loss of GGN Leads to Pre-implantation Embryonic Lethality in the Mouse

**Figure 2:**
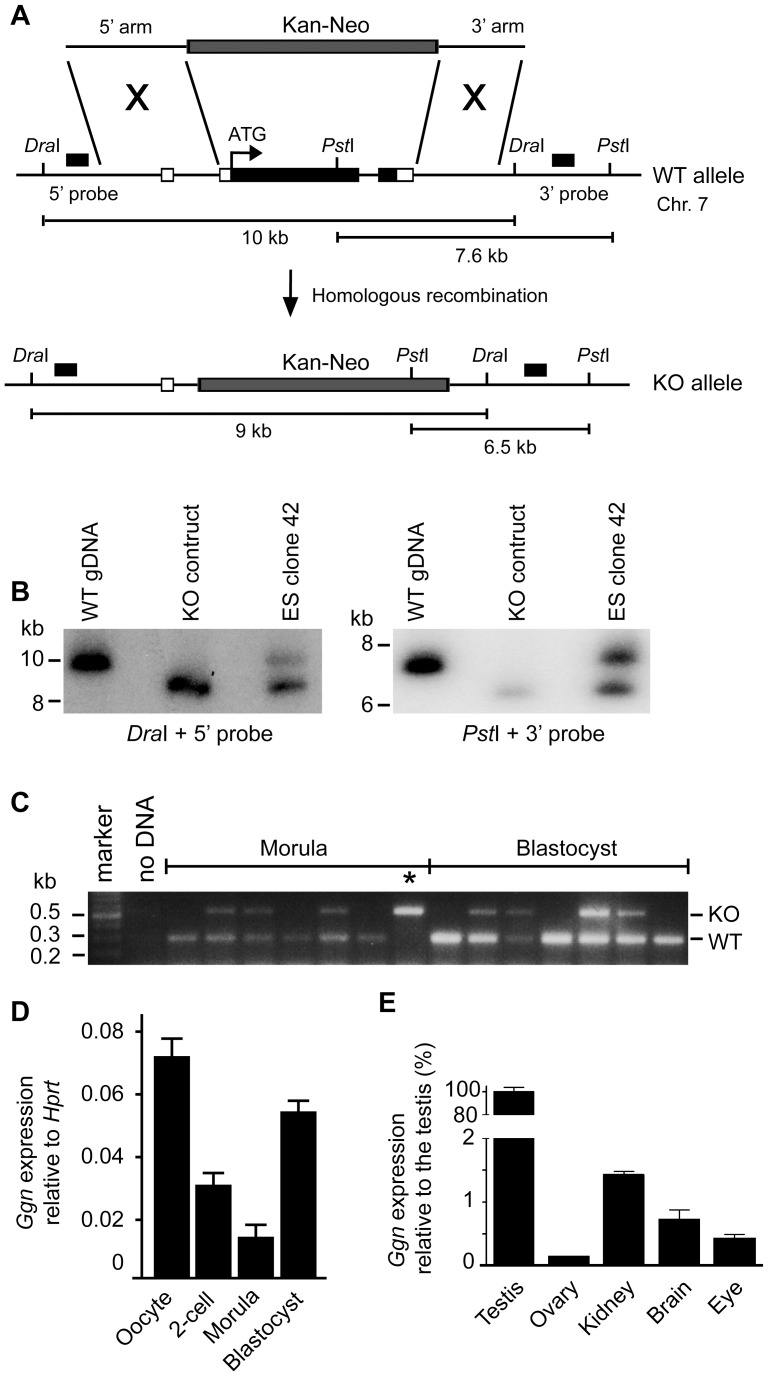
*Ggn* ^−/−^ embryos die prior to implantation. (**A**) Targeting strategy used for disruption of the mouse *Ggn* gene and for screening of the targeted ES clones (**B**) Southern blotting using 5′ and 3′ external probes. (**C**) Genotyping of pre-implantation embryos collected from *Ggn* ^+/−^ timed mating. *indicates a *Ggn* ^−/−^ embryo identified at morula stage of development. (**D**) *Ggn* is expressed in mouse oocytes and pre-implantation embryos. (**E**) *Ggn* is expressed at high levels within the adult testis and at a low level in the ovary and somatic tissues. All adult tissues were obtained from 10 weeks-old C57BL/6J mice. The y axis is truncated from 2–80%.

While the expression of GGN highlighted its potential role in male germ cell development, the *in vivo* function of *Ggn* has not been defined. As such we generated a *Ggn* null mouse line (Figure 2A). The strategy used to generate the *Ggn* knockout mice was to delete the protein-coding region of *Ggn* gene in all cell types. Targeted 129Sv ES clones were verified by Southern blotting using 5′ and 3′external probes (Figure 2B). Although the heterozygous knockout mice (*Ggn* ^+/−^) appeared grossly normal compared to wild-type littermates (*Ggn* ^+/+^), homozygous knockout mice (*Ggn* ^−/−^) were never found at weaning age (Table 1). To assess the potential for embryonic lethality, embryos from the *Ggn* ^+/−^ intercrosses were harvested for genotyping at various stages of gestation between days 7.5–13.5 *post-coitum* (E7.5–E13.5). No *Ggn* ^−/−^ embryos were observed at or beyond E7.5 (Table 1). These data, and the absence of any evidence of embryo resorption, suggested that *Ggn* ^−/−^ embryos died prior to implantation. Indeed, the genotyping of E2.5–E3.5 embryos collected from timed *Ggn^+/−^* intercrosses revealed that only 2% of *Ggn* ^−/−^ embryos survived to the morula stage (Table 1 and Figure 2C). An analysis of 2-cell embryos obtained by *in vitro* fertilisation (IVF) using oocytes and sperm from *Ggn* ^+/−^ mice however showed that *Ggn* ^−/−^ embryos were present in the expected Mendelian distribution (Table 1). Together, these results indicate that the majority of *Ggn* ^−/−^ embryos survive the first mitotic division, but all die prior to implantation. Thus, our data define the essential physiological role for GGN in the maintenance of embryo viability at the very earliest events of pre-implantation embryo development.

**Table 1:** Targeted deletion of the mouse *Ggn* gene resulted in pre-implantation embryonic lethality.

Age of progeny	Number analysed	Litter size(Mean±S.D.)	Genotype		
			Number of *Ggn* ^+/+^	Number of *Ggn* ^+/−^	Number of *Ggn* ^−/−^
3 week	175	7.9±2.1	52 (30%)	123 (70%)	0
E11.5–E13.5	48	9.5±2.0	13 (27%)	35 (73%)	0
E7.5–E8.5	48	9.7±1.7	14 (29%)	34 (71%)	0
E2.5–E3.5	45	not analysed	10 (22%)	34 (76%)	1* (2%)
2-cell IVF embryos	49	not analysed	12 (25%)	27 (55%)	10 (20%)

* indicates a *Ggn* ^−/−^ embryo identified at morula stage of development.

Next, we sought to determine if GGN is required for ES cell viability. Cells carrying two sets of the neomycin resistance cassette are more resistant to G418 than are those with a single copy of the cassette [19]. The two independently targeted *Ggn^+/−^* ES cells were subjected to clonal selection in the presence of a higher dose of G418 in attempts to generate *Ggn^−/−^* ES cells by gene conversion. No *Ggn^−/−^* ES cells were identified from 182 ES colonies examined, suggesting that GGN is required for the establishment of viable ES cells. The inability to isolate *Ggn^−/−^* ES cells in concert with the absence of *Ggn^−/−^* embryos beyond the morula stage strongly suggest that GGN is essential for cell viability.

### Ggn is Ubiquitously Expressed at Low Levels in Pre-implantation Embryos and Somatic Cells

In addition to high levels of expression in the testis, low levels of *Ggn* transcripts were detected in ovulated oocytes and pre-implantation embryos as determined by a quantitative RT-PCR (qRT-PCR) assay that detected *Ggn1*, *Ggn2* and *Ggn3* transcripts (Figure 2D). Further, we observed low levels of *Ggn* expression in the majority of adult mouse (10 weeks-old) tissues examined (Figure 2E). Thus *Ggn* is expressed more widely than previously anticipated, suggesting a role for GGN in the development of both germ and somatic cells. Indeed, this hypothesis is strongly supported by the embryonic lethality of the *Ggn* ^−/−^ embryos. The death of the vast majority of *Ggn^−/−^* embryos prior to morula and an absence by the blastocyst stage, is consistent with a loss of viability once maternal *Ggn* mRNA stores are depleted i.e. declined expression in morula (Figure 2D).

### Ggn^+/−^ Pachytene Spermatocytes Show an Increased Incidence of Unrepaired DSBs

**Figure 3:**
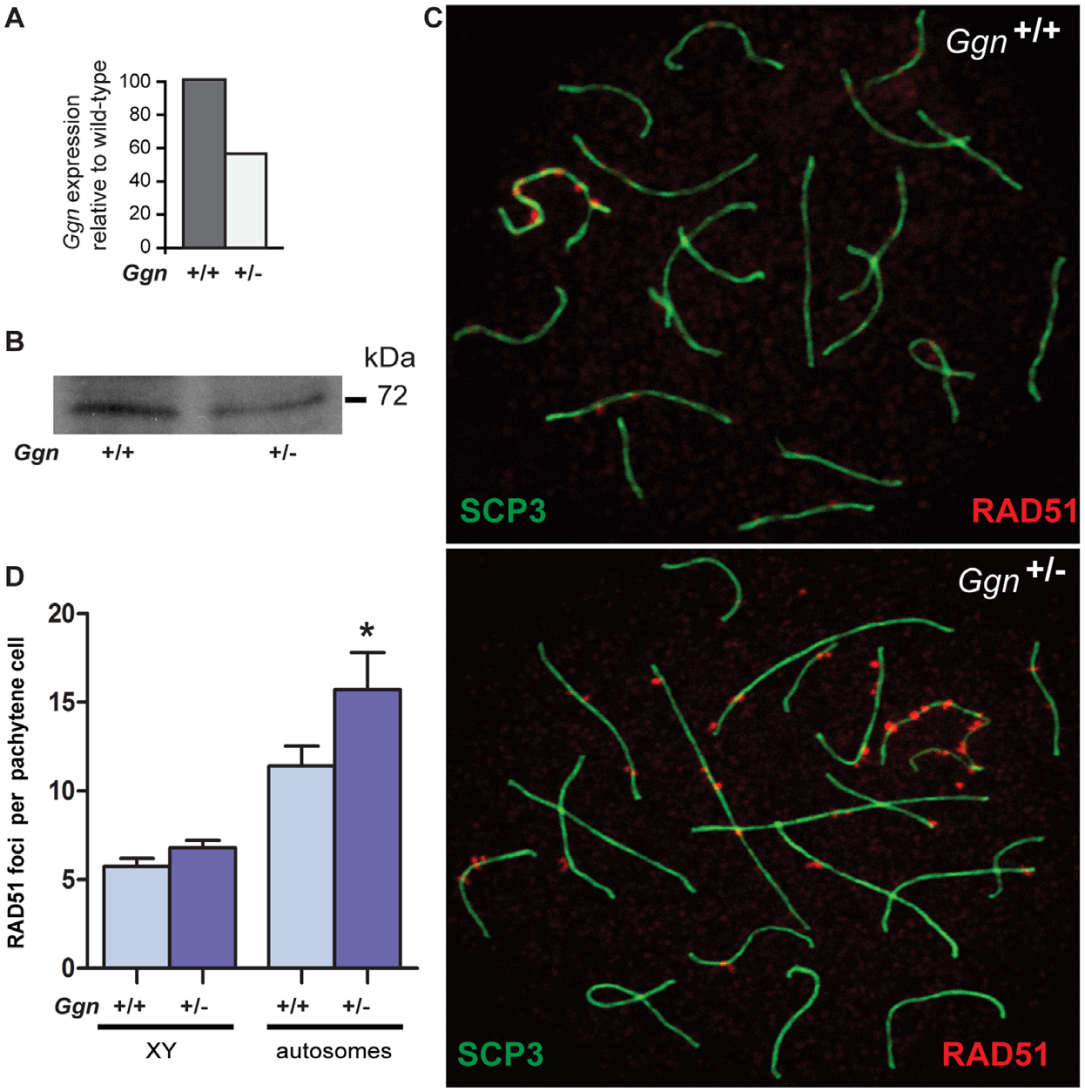
GGN haploinsufficiency resulted in compromised meiotic DSB repair. (**A**) qRT-PCR analysis and (**B**) GGN1 immunoblotting showed a reduction of *Ggn* transcripts and GGN1 protein in the *Ggn^+/−^* spermatocytes compared to that of *Ggn^+/+^*. (**C**) RAD51 foci in pachytene spermatocytes from the *Ggn^+/+^* (wild-type) and *Ggn^+/−^* (heterozygous knockout) mice. DSBs were visualised with a RAD51 antibody (shown in red), and stage of meiosis was marked with a SYCP3 antibody (shown in green). (**D**) RAD51 foci count per pachytene cell. RAD51 foci were counted from a total of 50 randomly selected pachytene spermatocytes from each of a total of 7 *Ggn^+/+^* and 7 *Ggn^+/−^* mice. Data are shown as mean ± S.E.M.

Next, we investigated whether GGN plays a role in DSB repair during male meiosis. However, this could not be explored on *Ggn* ^−/−^ male mice because of embryonic lethality, so we asked instead if meiotic DSB repair would be impaired by GGN haploinsufficiency in the *Ggn^+/−^* mice. To confirm haploinsufficiency, we performed qRT-PCR and GGN1 immunoblotting on purified spermatocytes from the *Ggn* ^+/+^ and *Ggn* ^+/−^ mice. We showed that *Ggn* transcripts in the *Ggn* ^+/−^ spermatocytes were reduced to about 60% of the *Ggn* ^+/+^ spermatocytes (Figure 3A). Consistently, GGN1 immunoblotting showed a reduction of GGN1 protein compared to that of *Ggn* ^+/+^ spermatocytes (Figure 3B).

SPO11-induced DSBs occur in leptotene and once formed are recognised by HR repair machinery such that by pachytene most DSBs are repaired [20]. To investigate whether the *Ggn* ^+/−^ spermatocytes have impaired DSB repair, spermatocyte chromatin spreads coupled with immunostaining were used. Spermatocyte chromatin spreads were prepared from *Ggn* ^+/+^ and *Ggn* ^+/−^ mice and double labelled with antibodies to the synaptonemal complex, SYCP3, as a marker of paired homologous chromosomes, and RAD51, as a marker of unrepaired DSBs. If GGN was involved in DSB repair during meiosis, then one possibility was that once the breaks were induced they would not be repaired. If this were the case we would observe more unrepaired breaks during pachynema. We analysed and quantified RAD51 foci on the autosomes and XY chromosomes of pachytene cells from the *Ggn* ^+/+^ the *Ggn* ^+/−^ mice (Figure 3C) (*n* = 7 mice per group, 50 pachytene cells counted per mouse, 350 cells per group). RAD51 foci per pachytene cell on the XY chromosomes were not significantly different between the *Ggn* ^+/−^ (6.78±0.44) and *Ggn* ^+/+^ (5.74±0.45) males (*P* = 0.06). However, we observed a statistically significant increase in autosomal RAD51 foci in the *Ggn* ^+/−^ males compared to that of *Ggn* ^+/+^ littermates (*P* = 0.04, 15.71±2.08 for the *Ggn* ^+/+^ males and 11.40±1.12 for the *Ggn^+/+^* males) (Figure 3D). Persistence of RAD51 foci in *Ggn* ^+/−^ pachytene spermatocytes indicated that meiotic DSB repair was impaired. Collectively these results suggest a role for GGN in DSB repair during male meiosis.

Many mouse models of *Fanc* protein deficiency exhibit fertility defects including those for *Fancl* [21], *Fanca* [22], [23], *Fancc* [24], *Fancg* [25], [26] and *Fancd2* [27]. Moreover, *Fanca* and *Fancd2* knockout spermatocytes showed elevated frequency of mispaired meiotic chromosomes [23], [27]. These observations highlight the critical role for the FA pathway in the maintenance of genome integrity in both somatic and germ cells. Herein we demonstrated that GGN1 as an endogenous binding partner of FANCL, FANCD2 and BRCC36 in the testis, and provide data to support a role for GGN in DSB repair during male meiosis. In order to definitely make such claims, however, it will be necessary to produce a testis-specific *Ggn* knockout model. Unfortunately this was not possible using the targeting strategy we have employed.

### A Potential Role for GGN in DNA Repair during Mitosis in Early Embryonic Development

The embryonic lethality of the *Ggn^−/−^* embryos is similar to that of mice lacking critical regulators of the DSB repair pathway, RAD51 [28], BRCA1 and BRCA2 [29], and ATR [30]. Several studies have demonstrated that the FA and BRCA pathways corporate DNA damage response and repair during mitotic cell division [31]. Mitotic errors in FA patients are common [32]–[34] and may be a consequence of unresolved DNA damage caused in the preceding S-phase. The FA pathway also plays a direct role in M-phase, where it prevents chromatid breakage resulting from unresolved sites of DNA crosslinking in the previous S-phase [34]. Moreover, it has been shown that double knockdown of BRCC36 and it binding partner BRCC45 compromised G2/M checkpoint in response to ionizing radiation-induced DNA damage [17]. These findings strongly support the critical role for the FA and BRCA pathways in DNA damage response and cell cycle progression in mitotic cells. The death of the vast majority of *Ggn^−/−^* embryos prior to morulae formation and an absence by the blastocyst stage suggest that only a few rounds of mitotic divisions occurred prior to cell death. These observations raise the possibility that the *Ggn^−/−^* embryos were incapable of repairing DNA breaks that occurred during early mitotic divisions and in turn may lead genome instability and cell death. Further investigations to define role for GGN in mitotic DNA repair during early embryonic development and in response to DNA damaging agents is required to delineate the mechanistic of action GGN plays in DNA repair.

In summary, we have shown that GGN is essential for the survival of pre-implantation embryos. The *Ggn* knockout mouse model described herein is unique and affects a relatively poorly understood aspect of pre-implantation embryo development. Identification of the pathway(s) through which the GGN protein acts should provide a better understanding of the biology of early embryo development. Data also suggests that in the postnatal testis GGN plays a role in DSB repair during meiosis.

## Materials and Methods

### Generation of the Ggn Knockout Mice

Animal experiments were approved by the Monash University and the University of Queensland Animal Ethics Committees. A 2.6 kb DNA fragment containing the entire protein-coding region of the *Ggn* gene was replaced with a 1.8 kb Kanamycin-Neomycin cassette (Figure 1A). Gene targeting was performed using the R1 ES cells (129X1/SvJ×129Sl) [35] purchased from Prof. Andras Nagy (Samuel Lunenfeld Research Institute, Toronto, Canada). The targeted ES clones were verified by Southern blotting (Figure 1B). Two independent targeted ES clones were injected into C57BL/6 blastocysts and the resulting male chimeras mated with C57BL/6 females to establish knockout mouse lines and subsequently backcrossed onto C57BL/6J for 12 generations. Both lines exhibited identical phenotypic defects. Genotyping of 3 weeks-old pups and post-implantation embryos was performed by multiplex PCR using two primer pairs: GGNa-Fw+GGNa-Rev and NeoR-Fw+NeoR-Rev (Table S1), whereby the wild-type alleles gave a band of 285 bp and the knockout allele gave a band of 537 bp. Genotyping of pre-implantation embryos was performed using a nested PCR strategy, whereby 1 µl from the first round of PCR amplification (using primers GGNa-Fw+GGNa-Rev and NeoR-Fw+NeoR-Rev) was used as a template for the second round of PCR amplification using primers: GGNb-Fw+GGNb-Rev and 2ndNeo-Fw +2ndNeo-Rev (Table S1). The wild-type *Ggn* allele gave a 360 bp product and the knockout alleles gave a 220 bp product.

### Defining the Timing of Ggn^−/−^ Embryo Loss

To obtain pre-implantation embryos from timed-mating, 6 weeks-old *Ggn* ^+/−^ females were superovulated as previously described [36] then mated with adult *Ggn* ^+/−^ males. The time of ovulation/fertilisation was defined as 13.5 hours post-hCG injection. Embryos were collected 1–3 days post-conception. *In vitro* fertilisation (IVF) was performed using *Ggn* ^+/−^ females and males as previously described [36].

### Generation of the Ggn^−/−^ Homozygous Knockout ES Cells

For clonal selection the two independently targeted ES cells were cultured in the presence of 2 mg/ml G418 (10-fold increase from the dose used for initial selection of the targeted ES cells) for 2 weeks. Surviving ES colonies were genotyped as described above.

### Ggn Expression Analysis

Quantitative RT-PCR (qRT-PCR) analysis was performed using TaqMan assays (Life Technologies) and normalised against *Hprt* (Hypoxanthine-guanine phosphoribosyltransferase). The TaqMan assay used for *Ggn* was Mm00717664_m1 and for *Hprt* was Mm00446968_m1. The *Ggn* TaqMan assay was designed to amplify the 3′ region of exons 2 and 5′region of exon 3 of the *Ggn1* transcript (NM_182694.2). This region is 100% identical to *Ggn2* (AF538033.1) and *Ggn3* (NM_182696.2) transcripts. To verify haploinsufficiency, spermatocytes were purified from *Ggn^+/+^* and *Ggn^+/−^* adult testes (10 weeks-old) using the Staput method as previously described [37]. qRT-PCR was performed as described above. Data obtained from the *Ggn* ^+/+^ spermatocytes was set to 100%.

### Immunoprecipitation

Testis protein extracts were prepared in lysis buffer containing 50 mM Tris-HCl, 150 mM NaCl, 1% NP-40, 2 mM MgCl_2_, 50 U/ml benzonase nuclease (Sigma), protease inhibitor cocktail (Calbiochem) and 5 mM sodium orthovanadate). The lysates were pre-cleared overnight with unconjugated agarose. 20 mg of affinity purified goat GGN1 antibody [8] and goat IgG were separately conjugated to resin using an AminoLink Plus Immobilisation kit (Thermo Scientific) as per manufacturer’s instructions. Equal amounts of testis extracts (4 mg) were added to the GGN1 or goat IgG column and incubated overnight at 4°C. Following extensive washing with PBS, bound protein complexes were eluted with 0.1 M glycine (pH 2.7) and separated on SDS-PAGE. Immunoblotting was performed using antibodies against FANCA at 2 µg/ml (ab97578, Abcam) FANCL at 2.5 µg/ml (ab94458, Abcam), FANCD2 at 1 µg/ml (ab2187, Abcam), FANCI at 2 µg/ml (ab74332, Abcam), BRCA1 at 0.2 µg/ml (sc646, Santa Cruz) and BRCC36 at 0. 25 µg/ml (ab115172, Abcam), and detected using ECL Plus (GE Bioscience). FANCL, FANCD2 and BRCC36 antibodies were used for reciprocal IPs as described above. Of these, FANCL antibody was unable to pull down FANCL protein. To confirm verify haploinsufficiency, 20 µg of spermatocyte protein extracts were loaded and probed with GGN1 antibody at 1 µg/ml.

### Meiotic Spread

Meiotic spreads were prepared as previously described from postnatal day 17–19 mice [38]. DSBs were visualised with a RAD51 antibody at 8 µg/ml (sc-8349, Santa Cruz), and progression through meiosis was marked with a SYCP3 antibody at 4 µg/ml (sc-74569, Santa Cruz). The first 50 pachytene spermatocytes for 7 *Ggn^+/−^* and *Ggn^+/−^* mice were photographed. Pachynema was defined as the presence of fully synapsed chromosomes i.e. no gaps. Foci on autosomes and the XY body were recorded separately. Statistical significance was determined using a student’s *t*-test was used to compare the means of two populations. P values <0.05 was used to define statistical significance.

## Supporting Information

Table S1 Primers used for *Ggn* knockout mice genotyping.
(DOCX)
