# Supplementary material for: Loss of GGN Leads to Pre-Implantation Embryonic Lethality and Compromised Male Meiotic DNA Double Strand Break Repair in the Mouse
Source: PLoS One. 2013 Feb 22;8(2):e56955. doi: 10.1371/journal.pone.0056955 (PMC3579931; doi:10.1371/journal.pone.0056955)
Supplement: Table S1 — Primers used for Ggn knockout mice genotyping. (DOCX) [file pone.0056955.s001.docx]

**Table S1: Primers used for *Ggn* knockout mice genotyping**

| **Primer name** | **Sequence (5’ to 3’)** |
| --- | --- |
| GGNa-Fw | TGTCCCTGCAAGTGCTACTG |
| GGNa-Rev | AAAGACCCAGGGTCAGCTTGGC |
| NeoR-Fw | GAAATCTCGTGATGGCAGGT |
| NeoR-Rev | TGCTCCTGCCGAGAAAGTAT |
| GGNb-Fw | GGGGTGTTATCCGTGAAGAA |
| GGNb-Rev | ACTTGTGGGTCAGCCTATGG |
| 2ndNeo-Fw | AATATCACGGGTAGCCAACG |
| 2ndNeo-Rev | GGATGATCTGGACGAAGAGC |
